# Automatic Emotion Recognition in Children with Autism: A Systematic Literature Review

**DOI:** 10.3390/s22041649

**Published:** 2022-02-20

**Authors:** Agnieszka Landowska, Aleksandra Karpus, Teresa Zawadzka, Ben Robins, Duygun Erol Barkana, Hatice Kose, Tatjana Zorcec, Nicholas Cummins

**Affiliations:** 1 Faculty of Electronics, Telecommunications and Informatics, Gdańsk University of Technology, 80-233 Gdańsk, Poland; aleksandra.karpus@pg.edu.pl (A.K.); tegra@eti.pg.edu.pl (T.Z.); 2 School of Computer Science, University of Hertfordshire, Hatfield AL10 9AB, UK; b.robins@herts.ac.uk; 3 Faculty of Engineering, Electrical and Electronics Engineering, Yeditepe University, Istanbul 34755, Turkey; duygunerol@yeditepe.edu.tr; 4 Department of AI and Data Engineering, Istanbul Technical University, Istanbul 34467, Turkey; hatice.kose@itu.edu.tr; 5 Center for Early Intervention for Children with Autism, University Children’s Hospital, 1000 Skopje, North Macedonia; tzorcec@gmail.com; 6 Department of Biostatistics and Health Informatics, Institute of Psychiatry, Psychology & Neuroscience, King’s College London, London WC2R 2LS, UK; nicholas.cummins@ieee.org; 7 Embedded Intelligence for Health Care & Wellbeing, University of Augsburg, 86159 Augsburg, Germany

**Keywords:** emotion recognition, affective computing, autism spectrum disorder, autism, systematic literature review

## Abstract

The automatic emotion recognition domain brings new methods and technologies that might be used to enhance therapy of children with autism. The paper aims at the exploration of methods and tools used to recognize emotions in children. It presents a literature review study that was performed using a systematic approach and PRISMA methodology for reporting quantitative and qualitative results. Diverse observation channels and modalities are used in the analyzed studies, including facial expressions, prosody of speech, and physiological signals. Regarding representation models, the basic emotions are the most frequently recognized, especially happiness, fear, and sadness. Both single-channel and multichannel approaches are applied, with a preference for the first one. For multimodal recognition, early fusion was the most frequently applied. SVM and neural networks were the most popular for building classifiers. Qualitative analysis revealed important clues on participant group construction and the most common combinations of modalities and methods. All channels are reported to be prone to some disturbance, and as a result, information on a specific symptoms of emotions might be temporarily or permanently unavailable. The challenges of proper stimuli, labelling methods, and the creation of open datasets were also identified.

## 1. Introduction

Autism spectrum disorder (ASD) is a lifelong, neurodevelopmental disorder that can occur to different degrees and in a variety of forms [1]. The fifth edition of the Diagnostic and Statistical Manual of Mental Disorders (DSM V) states that persons with ASD show deficits in social-emotional reciprocity, ranging from an abnormal social approach and failure of normal back-and-forth conversation to a total lack of initiation of social interaction [2]. The social communication challenge that faces individuals with autism is rooted partially in emotion recognition (ER) deficits that underline the difficulties in processing and interpreting socio-emotional cues [3]. Individuals with autism demonstrate impairment in social cognition that includes the identification of facial expression, face recognition, discrimination of faces, and memory for faces. As a result, individuals with autism often demonstrate increased stress and anxiety, abnormal perception of faces, and impaired processing of emotions [4].

This paper is part of the work of the Erasmus+ project EMBOA: *Affective loop in Socially Assistive Robotics as an intervention for Children with Autism* (https://emboa.eu/, accessed on 20 December 2021). The project aims to implement, evaluate, and develop guidelines into the feasibility of applying emotion recognition technologies in robot-supported intervention for children with autism in order to create an affective loop in child–robot interactions. The project combines three domains: autism therapy, social robots, and automatic emotion recognition [5]. Having said this to establish the context and motivation of the presented study, in this paper, we focus on automatic emotion recognition applied on children with autism, not only in the context of child–robot interaction applications, but with the purpose of robots perceiving emotions in mind.

The purpose of this paper is to report the results of a systematic literature review aimed at exploration of the state of the art in the automatic emotion recognition technologies applied to recognizethe emotions of children with autism. To be more precise, in the field of interest there are those studies which show how to automatically recognize emotions felt by the autistic children, not the capacity of children to recognize emotions in others. Following [6] we understand automatic emotion recognition as an interdisciplinary research field which deals with the algorithmic detection of human affect, e.g., anger or sadness, from a variety of sources, such as speech or facial gestures.

There are three studies (literature reviews) that we follow in our study [7,8,9]. In the study by Kowallik A. E. and Schweinberger S. R. [7], the authors review papers related to sensor-based social information processing. They focus on studies that use sensors to identify (diagnose) autism and to support intervention. The study does not focus specifically on emotion recognition (only three of the mentioned intervention papers are related to emotion recognition), although the listed modalities are the ones used in automatic recognition as well. In the study by Chaidi I. and Drigas A. [8], the authors present a literature review on both the expression and understanding of emotions in autism. They refer to the perception of emotions by children with autism rather than to the recognition of the emotions of the children. The study by Rashidan et al. [9] focuses on emotion recognition applied on children with autism. The study raises research questions regarding the stimuli used and the method of feature extraction, and might be considered as complementary to ours. Moreover, the reviews do not report challenges and recommendations for using the emotion recognition technologies, while this is an important part of our study. In our paper, whenever we refer to emotion recognition, we mean the automatic one, applied to recognizing emotion in children.

This paper presents a systematic literature review (SLR [10,11]) of automatic emotion recognition in children with autism. The paper uses the PRISMA (Preferred Reporting Items for Systematic Reviews and Meta-Analyses) [12] standard for reporting the study and is organized as follows. Section 2 describes the research methods used and the systematic literature review execution. Section 3 reports the quantitative and qualitative results. The results are followed by a discussion of research validity and an outline of challenges that future works might address.

## 2. Methods

A systematic literature review was used in the study as a methodological approach for capturing the state of the art in the domain of interest. The systematic method was chosen as the study aimed at finding key studies and performing the review with transparency and rigour that would allow replicating the study [10,11]. According to the PRISMA approach, the following steps were performed: setting up the research questions, defining the keywords, search string, and the inclusion and exclusion criteria, deciding on the search engines, performing the data extraction, a multiple-phase selection based on quality criteria and research questions, the final selection of papers and snowballing technique, and the extraction of the key findings. The design of the study is described in detail in Section 2.1, Section 2.2, Section 2.3, Section 2.4 and Section 2.5.

### 2.1. Research Questions

In the study, we aimed at the identification of the key previous studies, covering all aspects, technical and psychological, related to automatic emotion recognition applied on children with autism. We finished up with four research questions:

- RQ1: What emotions are recognized in children with autism in these studies?
- RQ2: Which observation channels are used in emotion recognition in children with autism?
- RQ3: Which techniques are used in emotion recognition in children with autism?
- RQ4: What techniques are used for multimodal recognition?

Regarding research question 1, the question covers the issues of distinct emotions that are automatically tracked in autism-related studies. The question also spans over the issue of emotion representation for the use of emotion recognition—whether they are yes–no labels or have a scale assigned. There are several popular models for the representation of emotions in affective computing [13]. The first one is Ekman’s model of basic emotions (happiness, anger, fear, sadness, surprise, and disgust), sometimes expanded with a neutral state if none of the six occur. In this model, the emotions could be treated as discrete (yes–no) or continuous [14,15]. Another popular model is a valence–arousal dimensional model of emotions, which represents an emotional state as a point in the plane of valence (positive–negative scale) and arousal (active–passive scale) [16]. Answering research question 1, we want to determine if those models are used or what alternatives are proposed in studies involving children with autism.

Regarding RQ2, by observation channel we mean a type of signal holding information on observable symptoms of emotional state that was used for emotion recognition. From the observation channels, one might extract modalities, e.g., facial expressions, body posture, skin conductance, or eye fixation areas. Modality is a type of information on a specific observable symptom extracted from the signal that is further analyzed to estimate an emotional state. Please note that one recorded channel might bring several modalities to analyze (e.g., both facial expressions and voice might be extracted from the video channel). A single modality might be obtained from multiple channels (e.g., facial expressions might be obtained from video channels or electromyographic sensors placed on the face). The question addressed in this paper is which channels and which modalities are used in the automatic recognition of emotions in children with autism.

Regarding RQ3 and RQ4, by technique we mean a technical data-processing method for the extraction of emotional state. One might find machine-learning algorithms, such as support vector machine (SVM) or neural networks, among frequent techniques for emotion recognition. Research question RQ4 was added because multimodal emotion recognition is frequently used to obtain more reliable and accurate results, and diverse fusion/integration mechanisms are applied. Therefore, in this study, we want to identify the recognition and fusion methods used in emotion recognition in children with autism.

### 2.2. Keywords

The keywords defined were grouped into the cluster related to emotions (emotion; affective; emotional; mood; affect; expression), related to children (children; child; young), and related to autism (autism; ASD; autism spectrum disorder; autism spectrum; ASC; autism spectrum condition; autistic; pervasive disorder). The final search query appears as follows:

 

(emotion OR affective OR emotional OR

mood OR affect OR expression)

AND (children OR child OR young)

AND (autism OR ASD OR ASC OR autistic

OR ‘‘pervasive disorder’’)

 

Please note that with a keyword “autism” we also cover phrases “autism spectrum disorder”, “autism spectrum”, and “autism spectrum condition”.

### 2.3. Inclusion/Exclusion Criteria

We settled on including in the SLR only original research and review papers written in English and published in journals or conference proceedings. We agreed to exclude papers written in other languages from the other phase of SLR for those databases where no language filter was available. Since emotion recognition in children with ASD is relatively new, and since we are interested in all psychological discoveries related to emotions in autism, no constraints on the publication date were made. We also decided to exclude short communications. These are our report eligibility criteria.

In addition to research questions and report eligibility criteria, we defined study eligibility criteria for studies to be included in the further stage of the SLR. We required that a paper concerns emotion recognition, and that there were children with ASD involved in the study. However, we also agreed to include some papers that do not satisfy these criteria (in the qualitative analysis only) if they brought some value, such as a well-described challenge or a guideline.

### 2.4. Search Engines and Search Strings

We decided to use seven scientific databases: ACM Digital Library, Elsevier Science Direct, IEEE Xplore, Scopus, SpringerLink, Web of Science, and PubMed. The first ones are the most popular scientific databases in technical sciences. The last one is important for scientists in medical and psychological domains and we chose to include it.

In the beginning, we performed an initial analysis to decide which search field we should use to obtain a feasible number of records for the SLR. We considered three options, i.e., all fields, title only, and topic/keywords only. Please note that some engines provide search by topic and some by keywords, which are not exactly the same, but were treated as equivalent in the study. The search was performed in two rounds—the first one dates October and November 2019 on technical databases and in January 2020 on PubMed, and the second round was performed in January 2022 to update the review with papers from years 2020 and 2021.

Each scientific database has its own search engine, resulting in a different query format. Thus, we had to slightly modify our search query to fit these requirements. For example, for IEEE Xplore, we had to add the field name to each keyword. The query for a title of a scientific paper is shown below.

 

(‘‘Document Title’’:emotion OR

‘‘Document Title’’:affective OR

‘‘Document Title’’:emotional OR

‘‘Document Title’’:mood OR

‘‘Document Title’’:affect OR

‘‘Document Title’’:expression)

AND (‘‘Document Title’’:children OR

‘‘Document Title’’:child OR

‘‘Document Title’’:young)

AND (‘‘Document Title’’:autism OR

‘‘Document Title’’:ASD OR

‘‘Document Title’’:ASC OR

‘‘Document Title’’:autistic OR

(‘‘Document Title’’:pervasive AND

‘‘Document Title’’:disorder))

 

Elsevier Science Direct allows the usage of only eight logical operators in a single query. Our search query contains fourteen ones. Therefore, we decided to split our query into six queries based on the first parenthesis and merge their results at the end of the searching process.

The number of obtained records for each database and search field in the initial search is presented in Table 1. For Elsevier Science Direct, we present two numbers for the title field. The main one is the sum of the number of results for all six queries which arise after splitting the original one. This also applies to other search fields. The one in parentheses is the number of records after removing duplicates.

Finally, we decided to use the title field because 1565 records is feasible to analyze, in contradiction to multiple-times more results from other fields. However, we are aware that this could lead to publication bias in our study. Some papers could be missing because they have not fitted into our selection criteria. On the other side, some studies could be reported in multiple papers and, thus, counted multiple times in the quantitative analysis.

### 2.5. Papers Selection and Key Findings Extraction

We settled on a manual selection of papers for their relevance to the eligibility criteria by title only. The papers should be evaluated on a three-point scale: 0—irrelevant, 1—somehow relevant, 2—strongly relevant. Four independent investigators recruited from the authors of this paper performed the tagging. Further decisions were made based on the sum of scores for taggers. Papers that scored 8 were taken to the next stage automatically. Papers that scored less than 4 were excluded automatically. Other papers with a score of 4–7 underwent screening by the abstract procedure. The Fleiss kappa coefficient was used to determine inter-rater consistency of tagging. Papers that passed the screening phase were analyzed in detail. We assumed that we could add additional papers during reading by noting out highly relevant papers from the bibliography that did not occur in the primary list of papers (according to the snowballing technique procedure).

We prepared forms in spreadsheets to extract the key findings in emotion recognition in children with autism. We were interested in several research issues related to automatic emotion recognition, i.e., which emotions are recognized in children with autism, which channels and techniques are used for emotion recognition, and how multiple modalities are handled. We also planned to analyze demographic data, including the number of children in the study (with ASD or typically developing), their gender, and age. We also wanted to note all challenges, recommendations, and other relevant observations. We agreed that a tagger could extend the forms, i.e., by new emotions or modalities that were not considered at the beginning of this study.

After we had read the papers and filled in the forms, we extended some of them which had too little information and repeated the process of the key findings extraction. For example, we extended a spreadsheet with information about children by adding vocabulary (wording) describing children. In some papers, authors used the expression “child with autism” while in others, “autistic child” was used. This procedure of repetition was to avoid selective reporting bias.

We decided to analyze the data by means of quantitative and qualitative analysis. For quantitative analysis, we used a simple count measure. We decided to unify some columns from the final spreadsheets, e.g., we grouped some emotion names under labels from the two emotion models: Ekman’s basic emotions or two-dimensional ones.

## 3. Results

The results of this study are presented in three subsections. In Section 3.1, a quantitative summary of the study execution is provided. Quantitative analysis results are described in Section 3.2. The analysis concerns the emotions recognized for autistic children and the channels and modalities used in this recognition process, and applied machine-learning techniques. Section 3.3 provides results from the qualitative analysis. It contains the wording about children and important guidelines and challenges that appeared in the analyzed papers.

### 3.1. Study Selection

We performed queries on selected scientific databases and obtained 1565 papers. After that, we unified the file format and merged all records to remove duplicates. We were left with 637 unique papers to perform an initial analysis on. We had to exclude one of them before tagging because there was no full-text version available anywhere.

**Figure 1:**
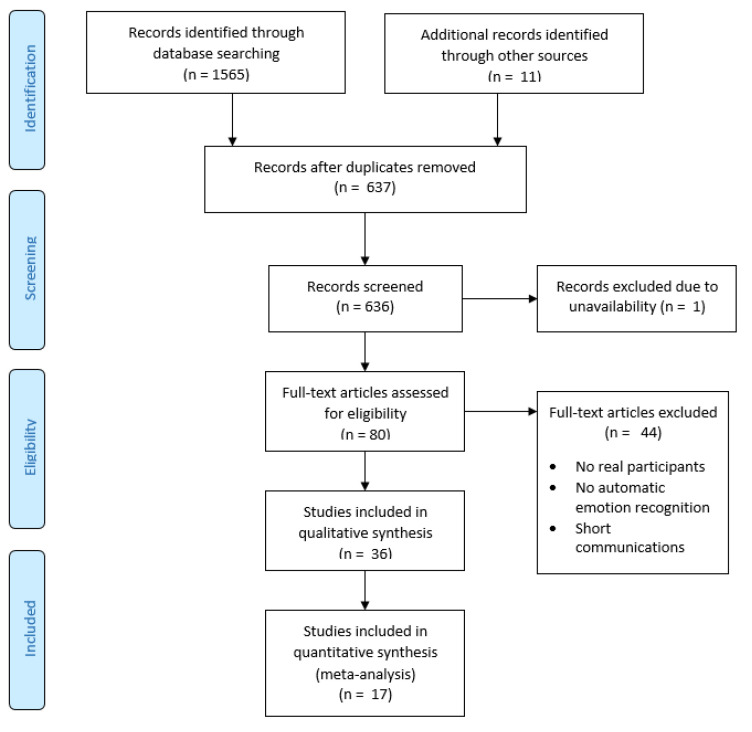
PRISMA flow diagram for the systematic review of the automatic recognition of emotions of children with autism.

First, the papers were manually tagged for their relevance by title only. The scale was: 0—irrelevant, 1—somehow relevant, 2—strongly relevant. The inter-rater consistency for the four taggers measured with Fleiss’ kappa coefficient was equal to 0.543. The sum of scores for the four taggers was calculated, and further decisions were made based on the total score. A total of 29 papers that scored 8 were taken to the next stage automatically; a total of 308 papers that scored less than 4 (score 3:60; score 2:43; score 1:47; score 0:158) were excluded automatically, while 299 papers that scored 4–7 (score 7:39; score 6:36; score 5:89; score 4:135) underwent screening by the abstract procedure. After the second tagging, we qualified 40 papers to the next stage, finishing with 69 papers for reading. We have identified 11 additional papers by the snowballing technique. From 80 papers, only 36 were qualified for detailed analysis and reporting. Another 44 papers were excluded after reading because there were no real participants or automatic emotion recognition. At this stage, we also excluded some short communications. Results from this phase of SLR are presented in Figure 1.

We identified two studies that were reported in multiple papers: one in two [17,18], and one in three papers [19,20,21].

**Figure 2:**
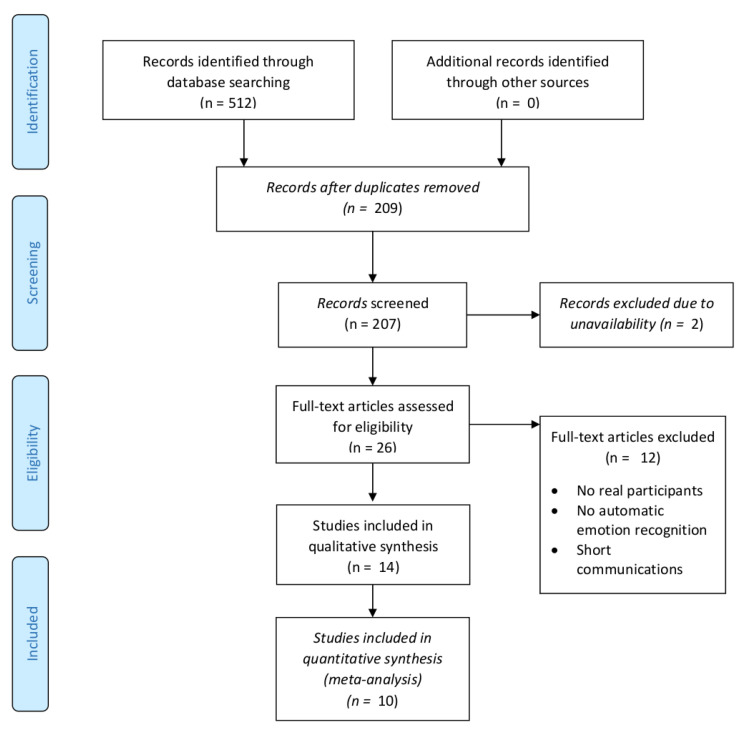
PRISMA flow diagram for the systematic review of the automatic recognition of emotions of children with autism in January 2022.

In January 2022, we performed additional searches on each scientific database to fill in the gap between the first search and a current date. We identified 512 papers. After removing duplicates, we were left with 209 papers for additional analysis. We repeated the same procedure as in the first search. Manual tagging by title was performed by four independent experts whose agreement measured with Fleiss kappa was equal to 0.607. After screening by title phase, we were left with 57 papers for the next phase. A total of 15 papers which obtained scores equal to 8 were taken to the full-text read phase automatically. Others had to pass a screening-by-abstract phase, in which we identified 15 relevant papers to include in the analysis. However, two of them were unavailable and two were in languages other than English (we agreed to include only papers written in English). Thus, we finished with 26 papers for the full-text read phase. After the last phase, we obtained 14 papers to include in the qualitative analysis and 10 papers to include in the quantitative analysis. Results from this phase of SLR are presented in Figure 2.

### 3.2. Quantitative Results

In this subsection, the quantitative analysis is presented. First, we present a discussion of notions (words) that are used to describe emotions in diverse studies in Section 3.2.1. Then, the quantitative analysis related to the discrete and dimensional representation of emotions being recognized is shown in Section 3.2.2.

Another part of the analysis concerns used channels registering various modalities in the process of emotion recognition (Section 3.2.3). Additionally, in this section, we present a cross-section of channels used and emotions recognized.

Next, the techniques of emotion recognition were analyzed to address the 3rd and 4th research questions. In Section 3.2.4, the analysis concerns machine-learning techniques used in the process of emotion recognition. These machine-learning techniques are analyzed versus the emotions being recognized and the channels used in the recognition process.

Section 3.2.5 presents whether a unimodal or multimodal approach is applied for obtaining emotion recognition results. For the multimodal approach, the fusion strategies are analyzed. The applications of these strategies vs. recognized emotions, used channels, and machine-learning techniques are presented.

#### 3.2.1. Emotions Recognized

In psychological research, there is no unambiguous definition of human emotion. However, a discrete emotions concept is widely accepted. There are multiple models to represent emotions. Analyzing papers that deal with emotion recognition, we have to address the issue of how emotions are distinguished and named in those papers. Some observations that influenced our analysis follow:

1. Most of the papers use two emotion models: Ekman’s basic emotions (joy, anger, fear, disgust, sadness, surprise) and/or a two-dimensional model of valence and arousal;
2. For Ekman’s basic model, papers use a subset rather than the complete set of emotions;
3. Papers use different wordings to describe the emotions, and additional attention should be paid to the meaning of those in a particular study;
4. The set of emotions resulting from the recognition process is not limited to the ones described by these two models. Thus, all the emotions recognized within the studies described in the analyzed papers were grouped into some subsets and further discussed;
5. Some notions used in the papers can be treated as moods or mental states, but all of them were included in the analysis. Emotions are analyzed in two ways: separately and in groups;
6. All notions other than dimensional representation are treated as discrete emotions.

Table 2 presents the various notions of emotions used in the studies. The emotions were grouped regarding their similarity.

There are studies that treat attention, engagement, involvement, and boredom as emotional states. They are more like mental states; however, they are emotion-related and were used in the context of emotion recognition in the analyzed papers.

There is a group of studies analyzing fear-related emotions; however, apart from the word “fear”, the words “anxiety” [20,22,25] and “trepidation” [37] were used. As the concepts differ, in further analysis for using basic emotions, we count papers that use the “fear” word only, and while we count all of them, they are referred to as fear-related emotions.

There is a number of papers for joy-related emotions, using terms of ‘joy’, ‘liking’, ‘happiness’, ‘smile’, and ‘relaxation’. Relaxation was added to that group, as in study [37], the recognition method applies both for happiness and/or relaxation altogether. The notions of “liking” and “happiness” are used in the “joy” meaning in the analyzed papers. There were more notions that refer to positive emotions (see Table 2). Thus, when analyzing occurrences of basic emotions, all papers where “liking” and “happiness” occurred are counted in the analysis for the “joy” emotion. A smile is not an emotion; therefore, paper [40] was not counted as recognizing joy.

Apart from the emotions presented above, calmness (1), hunger (1), and nerves (1) are also recognized in those papers. Hunger and nerves are both recognized in the same study [37], and calmness is recognized in [36].

A quite-different approach from any other works is presented in [41], where the negative, positive, and neutral affect state is recognized—the whole group of emotions is recognized, not only the specific one.

#### 3.2.2. Discrete and Dimensional Emotion Models Used

The quantitative analysis for papers describing the recognition of emotions comprises the emotions in the Ekman model (enriched with neutral emotion) in Table 3, as well as the two-dimensional model.

As mentioned above, Ekman’s basic emotions are frequently combined and recognized together, however rarely the complete set of emotions is used. Joy (also named as happiness) is the most frequently recognized emotional state (21 times). Rarely recognized states are disgust and surprise (both only 7 times). The basic emotions are also combined with a neutral state.

The combinations of basic emotions that occurred in the analyzed papers are shown in Table 4.

**Figure 3:**
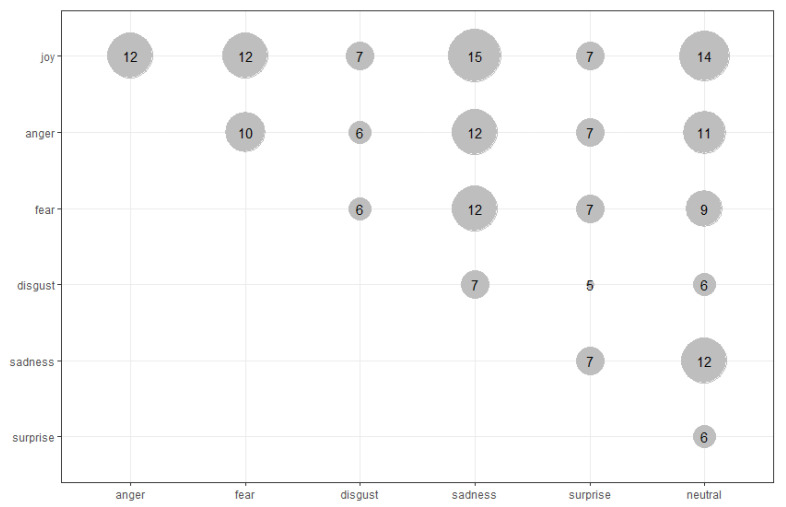
Co-occurrence of basic emotions in recognition studies.

In some papers, only two-class recognition was performed. In those papers, joy is recognized as a class combined with no-joy in three papers, with a neutral state in two papers, and with sadness in two papers. The other basic emotions are occurring combined with joy. The complete set of basic emotions (as proposed by Ekman) is rare—appearing five times (in four studies combined with the neutral emotional state). In [41], a neutral state is recognized and this emotional state is combined with positive and negative emotions (not the Ekman’s ones). The co-occurrence of specific emotions is presented as a bubble chart (Figure 3).

An interesting study combines joy and sadness with hunger, trepidation, relaxation, and nervous states [37]. Regarding the dimensional model of emotions, it is represented in seven papers—valence and arousal are always recognized together [17,23,34,35,42,43]. Some studies added a dimension of dominance [18] or analyzed arousal only [44], but they were excluded from the quantitative analysis as they were preliminary and provided no validation with children with autism.

There are studies that deal with compound emotion concepts [45,46]. A compound emotion occurs when a person expresses a state that is a combination of basic emotions. The studies were not included in the quantitative analysis, because they deal with recognition of meltdown crisis in autism rather than the recognition of a specific emotional state (basic or compound); however, both studies emphasized that human emotions might include far more than six basic emotions. During meltdown crises in autism, the following compound emotions were observed: happily disgusted (which is not really joyful, but rather nervous smiling), angrily disgusted, fearfully disgusted, sadly surprised, fearfully surprised, angrily surprised, disgustedly surprised, fearfully angry, and sadly angry [45,46].

#### 3.2.3. Channels Used for Emotion Recognition

When analyzing inputs used for emotion recognition, it is important to define this analytical process on the generic level and distinguish between life activities, observation channels, and modalities. The process of emotion recognition is analyzed with respect to **life activities**, i.e., conscious and unconscious actions of a human body, which generate a specified symptom of an emotional state that can be further analyzed in the process of emotion recognition. The following life activities were analyzed in the selected papers: various types of movement, a sound made by a human, and physiological activities such as heart activity, unconscious muscle activity, respiration, and thermal regulation. The activation of the human body’s nervous system induces changes in life activities, and those might be interpreted as symptoms of emotions.

The life activities might be recorded via **observation channels**, which are mediums for the registration of a signal holding information about observable symptoms. The channel refers to a type of signal obtained rather than a physical medium. The channels that were used in the studies of emotion recognition in children with autism include RGB video, depth video (Kinect mainly), images, audio, ECG (electrocardiography), BVP (blood-volume pulse), chest size, EMG (electromyography), fMRI (functional magnetic resonance imaging), EDA (electrodermal activity), and temperature.

The life activities generate **modalities**, which are understood as a type of information observable and used as a proxy for emotion recognition. In our study, modalities are grouped according to life activities:

- movement: facial expressions, body postures, eye gaze, head movement, gestures (also called hand movements), and any other not-previously classified motion;
- sound expressions: vocalizations, the prosody of speech;
- heart activity: heart rate, HRV (heart rate variablity);
- muscle activity not related to movement: muscle tension;
- perspiration: skin conductance;
- respiration: intensity and period, ECG;
- thermal regulation: peripheral temperature;
- brain activity: neural activity.

There are also some other modalities not mentioned here—only modalities identified for the need of this paper are listed.

Further, each modality could be paired with the observation channel that it was extracted from. Thus, one modality can be observed using various channels and one channel can contain information about more than one modality. In Table 5, the papers are assigned to the life activities, modalities, and channels used in the process of emotion recognition described in the analyzed studies.

There are some usual combinations of channels and modalities extracted from them (such as facial expressions extracted from RGB video), but Table 5 shows that there is a diversity of modalities and channels used in studies of emotions of children with autism.

Interestingly, a lot of attention is paid to physiological signal measurements and speech analysis. The reason for that might be assigned to the atypical expressivity of children with autism in terms of mimicked expressions, which was observed in the psychological studies multiple times. Moreover, at least some children with autism would not use active speech as well. Therefore, other vocalisations and physiological responses of the nervous system are more frequently used.

Another analysis performed was the collation of analyzed modality and the emotion recognized from it. In the consequent Table 6 and Table 7, the studies are assigned to a cross-section of recognized emotion and modality. Table 6 is for Ekman’s six basic emotions plus neutral state and a two-dimensional model, while Table 7 is for other related groups of emotions.

Joy/happiness versus a neutral state was the most commonly recognized of almost all types of modalities. Quite interestingly, while physiological signals are observed and processed, joy was almost exclusively the emotion recognized from it (see Table 6). There is a group of papers that concentrates on emotional states combined with mental states (attention), and these papers analyze physiological signals mostly (see Table 7).

There is another very interesting study that uses an atypical observation method for recognition of affect in children with autism, namely, the analysis of children’s drawings [47]. The authors claim that it is possible to analyze the colors, closeness of objects, line continuity and thickness, and stroke characteristics captured by a specific mobile application in order to recognize a child’s emotional state with over 59% accuracy for five classes. Although the applicablity of the approach is limited to this single activity only, the approach is an interesting alternative to measuring symptoms in children.

#### 3.2.4. Machine-Learning Techniques Used for Emotion Recognition

Knowing which channels were analyzed, various machine-learning techniques were identified, such as the ones used for emotion recognition, ie., neural networks (NN), fuzzy c-means clustering (FC-M), support vector machine (SVM), random forest (RF), hidden Markov model (HMM), linear discriminant analysis (LDA), k-nearest neighbors (k-NN) and fuzzy logic (FL). Table 8 presents which machine-learning technique is used for recognizing the specified emotions in both models as well as the identified groups of emotions.

In two papers [30,37], there is no automatic emotion recognition method described; however, the emotional analysis presented in these papers can be a base for building such methods. Thus, these two papers are not included in the tables presenting machine-learning techniques used in the automatic emotion recognition methods. In [35], emotions are recognized by an external vendor-provided solution (the machine-learning technique that was used is unknown); thus, this paper is not included in Table 8 and Table 9.

#### 3.2.5. Analysis of Approaches Used in Emotion Recognition for Multimodal Processing

When one or more modalities or channels are analyzed together, the discussion must also concern the applied approach for multimodal processing. There are two main techniques: early fusion and late fusion. Early fusion combines features extracted for various modalities and channels, and these merged features are further analyzed using statistical or machine-learning techniques. On the other hand, by using late fusion, it is assumed that the emotions are recognized from each channel for the specified modality separately, and then the new emotion is determined using a machine-learning technique basing on previously recognized emotions. Additionally, there is also a hybrid fusion approach. This approach combines early fusion with late fusion. As some features may be combined as an input for machine-learning techniques (early fusion for selected groups of features), the late fusion is also applied afterwards.

Two unimodal approaches are presented: only one channel for one modality is analyzed, marked as a single-channel-only approach, or more than one channel, or modality is analyzed but independently from other modalities and channels, which is marked as a multiple-channels separately analyzed approach. This approach is also marked when more than one channel is analyzed but only one channel is used to recognize the specific emotion. It is worth noticing that in two papers [30,37], there is no automatic recognition method described; however, emotions are analyzed separately for one channel only. Thus, these analyses are treated as unimodal analyses (single channel only).

The analysis presented in Table 10, Table 11, Table 12, Table 13, Table 14 and Table 15 shows which approach for unimodal/multimodal processing is applied in which studies. The analysis of modalities and channels analyzed together is also presented. In Table 10 and Table 11, the unimodal/multimodal analysis is presented with correspondence to emotions (Ekman’s model and two-dimensional model—Table 10, and identified groups of emotions—Table 11). In Table 12, the unimodal/multimodal approach is analyzed versus modalities and channels, and in Table 9, the unimodal/multimodal approach is analyzed versus machine-learning techniques. In Table 13, the number of papers where various modalities are analyzed together exclusively (no other modalities are taken into consideration) is shown. Table 14 presents the number of occurrences of specific modalities’ pairs recognized together (it does not matter if any other modalities are processed as well). There is no table showing the number of papers presenting the channels used within the emotion analysis process exclusively (no other channels are taken into consideration). This is caused by the fact that, for each paper, the set of channels is rarely the same, i.e., [19,22] used EMG, ECG, and EDA. Table 15 presents the number of occurrences of specific channel pairs analyzed together (it does not matter if any other channel is analyzed as well).

### 3.3. Qualitative Results

Apart from quantitative analysis, 50 papers were analyzed in a qualitative manner (36 from the first and 14 more from the second round). We were looking for recommendations and best practices in terms of techniques used, study preparation, and conduct.

#### 3.3.1. Participants

We have studied papers that referred to a group of children with autism. We noticed that papers differed in vocabulary (wording) when describing children. In some papers, authors used the expression “child/children with autism”, while in others, the expression “autistic child” was used. The latter one is deprecated, as the tendency now is to put “a person first”. Moreover, papers differed in the reference to a control group—“typically developing children” or “neurotypical children” are preferred over “normal children”. More vocabulary items are presented in Table 16, where the number of papers, in which the specific notion is used and the number of all occurrences of the notion, are depicted. These values were calculated automatically in Python script which transformed .pdf files to text using pdfplumber library. Please note that if a paper defines a shortcut, e.g., NT for neurotypical children, those occurrences are not counted. For generating the table, we used papers [17,19,20,21,22,23,24,25,26,27,28,29,30,31,32,33,34,35,36,37,38,39,40,41,42,43,44,45,46,47,48,49,50,51,52,53,54,55,56,57,58,59,60,61,62,63,64,65,66].

Approximately half of the papers that had emotion recognition and children with autism in the title reported only preliminary studies. Some researchers referred to children with autism as a target group of their solution in their studies, but did not report the children’s actual measurements. They reported results for adults, a group of typically developing children, or they did no validation at all. Two studies used previously generated datasets of recordings or measurement data [18,67]. Those datasets were not recorded for children with autism, but were studies aimed at future applications to that target group. There is a dataset that contains recordings of children with autism. It was created by the deENIGMA project and might be used in future research as it is openly available [23].

Only 17 studies provided validation with children with autism. The observations regarding participants are as follows.

The participants’ groups were relatively small—most of the studies involved 1–7 children, and a maximum of 20 participants was reached in 2 studies; however, the number of children with autism was 14 at most. There was one study using a dataset produced by the deENIGMA project, with recordings from 35 children with autism. In only six studies, children with ASD were paired with a group of typically developing children, while the other studies involved children with ASD only. In most of the studies, children with autism were treated as a homogeneous group with no further distinction between high- and low-functioning ones. For only six papers, the functioning of children was provided, and in four of those, only high-functioning children were included in the study. Most of the studies reported the age of participants, which varied from 2 up to 20 years (chronological age). Four out of seventeen studies did not provide age. Most of the studies reported the sex of the children, and among children with autism, girls were underrepresented (for the studies that provided sex, only 18% of participants were girls). Recommendations regarding the construction of participants group include:

- construct the control group according to developmental age and/or intelligence factor rather than the chronological age of ASD group;
- making an openly available dataset of recordings is a highly recommended approach, as it fosters further research;
- regarding the construction of participant group, it might be hard to balance girls’/boys’ inclusion due to population skewness (autism is diagnosed in boys more frequently); similarly, it might be hard to balance the group regarding low-/high-functioning children, as low-functioning children might refuse to participate more frequently and their participation also depends on the type of activities under study.

Additionally, the other systematic review study reports the following as future works: widen the age range (in two studies), varied demographics (four), larger sample size (four), prior ASD clinical test (one), repeat to the same age group (one) [9].

As autism deficits occur in a variety of forms and levels in individuals, the challenge of generalizability of the results remains inherent in all of the studies. Therefore, regardless of the group construction, in each study, it would be valuable to provide information on participants that is as detailed as possible, including, at least: chronological as well as developmental age, gender, and level of functioning.

#### 3.3.2. Stimuli and Tagging Practices

There were multiple strategies used in the studies for evoking emotions (stimuli) and tagging them. Regarding the stimuli, both evoked emotions and natural interaction approaches were used, and the challenge of collecting a proper training set of data from children with autism was raised [51].

The most common stimuli used in the studies included pictures and video resources. However, a study [37] on galvanic skin response revealed that pictures are not suitable stimuli for evoking emotions in children with autism.

In another study, eye gaze patterns were analyzed as a reaction to stimuli of videos containing human faces [50]. Main findings include:

- There are differences in reactions to positive and negative emotions presented in stimuli;
- Live videos of natural, real humans are good stimuli for tracking reaction patterns in children with ASD;
- There are differences in fixation duration time regarding different areas of interest (eyes, mouth) between children with ASD and typically developing children.

Although video stimulus was the most frequently used one, other studies tried: serious game approach [65], computer-based intervention tools [21], and observation of natural human–robot interaction [20,31].

The assignment of a proper (accurate) label to the data gathered is another challenge in emotion recognition in general, not only in autism. The internal emotional state (ground truth) is hard to determine in a continuous manner, sometimes even for a person who experiences it. Several strategies might be used for labelling the data. In [42], skilled therapists annotated recordings from multiple therapy sessions, and that was the most common practice. Another approach that uses subjective reports of the affective states from caregivers was introduced [22], and was compared to therapists’ reports with a consistency of approximately 83%. In a single study of a human–robot interaction loop [20], self-reporting was used; however, this was combined with therapists’ evaluations as well. Self-reporting in children with autism was only partially consistent with tagging by therapists. Therapists’ reports were taken as a “ground truth” for classification. The authors state that due to the deficits in communication skills in children with autism, the “classic” methods for emotion tagging are hard to apply. They recommend that for the enhancement of the reliability of tagging, both a clinical observer and a caregiver who knows the participant shall be included in the study.

#### 3.3.3. Modalities and Their Processing in Autism

Section 3.2.3 summarizes quantitatively the modalities (observation channels as well as emotional symptoms derived from them) used in emotion recognition in autism. This subsection adds some more observations on the challenges encountered in the studies regarding the modalities. The challenges encountered in the studies fall into three categories:

- the measurement procedure or environment might be disturbing for a child with autism;
- the data obtained are not of high quality (hold no significant information on a child’s symptoms of emotions);
- the obtained modalities are hard to analyze due to atypical patterns of symptoms.

Regarding the challenge of the measurement procedure, the more we want to measure in the study, the more complex the procedure becomes, and all the devices introduced in the environment might be disturbing for a child. Children with autism usually like repeatable, familiar environments, and all intrusions, including devices put on the body, devices in the room, and extra people, might cause a refusal to participate or might be a reason to behave differently. For example, a study on smile recognition [40] utilizes wireless EMG put on a child’s face. The study reports that 70% of children agreed to wear facial EMG devices, which means that the data simply could not be obtained for some children. The study did not measure the influence of wearing the device on child behavior. Moreover, some studies suggest using multiple channels and signals to increase reliability [51]; however, one must be aware of the trade-off between creating a more complex environment and having more reliable measurements. Some authors strongly recommend the familiarization stage (to measurement conditions and procedures) [52].

The second challenge is that even though one will record the session using devices, the data will not contain the information sufficient to draw conclusions on symptoms of emotions. This applies, for example, to eye gaze tracking, which is hard to calibrate, but also to other modalities. For example, [50] describes important observations concerning sensors and technologies that can be used in automatic emotion recognition:

- children with ASD had significantly lower amplitudes of respiratory sinus arrhythmia and faster heart rates than typically developing children at baseline, suggesting lower overall vagal regulation of heart rate;
- a large percentage of children with autism had abnormally high sympathetic activity, i.e., skin conductance response;
- it is difficult to employ eye-tracking technologies with lower-functioning children because the calibration and data-collection processes require the child to sit still.

Another study [35] reports obstacles that might be encountered while analyzing facial expressions from video recordings. The obstacles were classified into activity-based, child condition-based, and setup-based ones, which is an important distinction. The activity-based ones are those that result from the scenario of interaction—some activities that would require movements of the head, hands, and body might naturally influence recording symptoms of emotions. Regarding the child condition-based challenges, a child with autism might exhibit refusal or other atypical behaviors, not fitting into the planned interaction scenario or the technical setup. Regarding the setup-based ones, authors report the limitations of using a camera placed in a robot eye–a robotic turning head as a part of the scenario or robot hair that occluded the scene in some recordings were reported [35]. Another study reported possible future works as: all-day recordings, fixed subject distance, and the limitation of subject movements [9]. While one might optimize the emotion setup to get as accurate a recording of emotional symptoms as possible, some of the obstacles are inevitable. It is important to emphasize that it should be emotion recognition technology that follows the child and the designed interventions, not the child and interventions adjusted to fix emotion recognition setup problems.

The third challenge refers to atypical patterns of emotion expression by children with autism. Paper [62] summarizes the findings on autistic individuals’ facial expression of emotions. Three macro-areas of equal height from forehead to chin—upper, middle, and lower face—are studied. Significant differences between high-functioning autism and typically developing participants for disgust and sadness can be found in all three facial areas, for joy in the upper and lower face, and for surprise in the lower face. No significant differences were found for anger or fear.

In one study [37], galvanic skin response data were compared between children with autism and typically developing ones. The study revealed that children with autism have more irregular patterns of skin conductance physiological signals. Adding to this, [51] points out two interesting questions:

1. How will the children’s emotions be displayed (in autism)?
2. Does emotional affect vary with the severity of the disorder? How can this be accounted for and by the model?

Another study of cross-modal coordination of emotion expressions reveals that the coordination is lower in the ASD group when compared to neurotypical children [66]. Children with ASD produce the same level of emotional facial expressions and speech at the same levels of intensity as typically developing children, but facial and vocal expressions are less coordinated with each other. According to the literature review summarized in the same paper, that cross-modal coordination applies also to facial expressions versus gestures. It is also reported that children with ASD exhibit atypical timing and synchrony of movements of different facial regions, reduced intensity of upper face movements, reduced variety of facial movements, and more ambiguity, as expressions for positive and negative valence do not differ as they do for the typically developing peers. The observations were confirmed by another study [38], which reported less synchrony of motions between facial expressions, less complex facial dynamics, and more ambiguity. The deficit in facial expression was independent of emotion type (happiness, anger, sadness, and neutral state were included).

Out of the life activities and modalities that are used as proxy symptoms for emotion recognition, all are prone to some disturbance. Eye gaze is hard to calibrate, as it requires child cooperation and sitting still. As emotion processing and expression is a deficit in autism, facial expressions might be disturbed and misleading. Some children are non-verbal, which limits the analysis of vocal clues of an emotional state. In addition, regarding physiological signals, the studies revealed atypical patterns.

#### 3.3.4. Applications

Once symptoms of emotions are captured, and the emotion classifiers are trained, one might consider applying them in various forms in real-life situations. Most of the studies analyzed are conceptual or laboratory-based ones, and they refer to therapy and/or diagnosis of autism as their future application areas. However, some contain more practical observations referring to real-life applications of emotion recognition in autism.

Paper [21] raises the challenge of how to make the computer-based ASD intervention tools affect-sensitive by designing therapist-like affective models of the children with ASD based on their physiological responses. The study proposed two computer-based cognitive tasks to elicit the affective states of liking, anxiety, and engagement that are considered important in autism intervention.

The paper [65] summarizes a study that acquires physiological signals during therapeutic sessions supported by interactive “serious games”, and correlates the autonomic nervous system response to the engagement of the child during socio-cognitive tasks for an evaluation of the treatment effect and for the personalization of the therapy. A wearable chest belt for electrocardiographic (ECG) signal recording was used in the study. Another study examined facial expressions during attention tasks [48].

The main aim of [26] was to demonstrate if computer vision-based approaches for facial feature analysis could help to understand emotional behaviors in children with the interesting perspective of introducing a computational approach for the diagnosis and assessment of autism spectrum disorders.

The paper [44] describes CaptureMyEmotion, an app for smartphones and tablets that uses wireless sensors to capture physiological data and facial expression recognition to provide a personalized interface to help children with autism identify and understand their emotions. CaptureMyEmotion enables children with autism to capture photos, videos, or sounds, and identify their emotions while taking the picture. While a child’s self-portrait is taken, the app measures the arousal and stress levels using wireless sensors.

Among the real-life application areas, interaction with social robots is a frequently represented one in the analyzed subset of papers. The study [23] processes multimodal data (audio, video, and autonomic physiology) to estimate affect and engagement in human–robot interactions. The study introduces interesting recommendations regarding unobtrusive sensing, perception of changes by a robot, and modulation of the robot’s behavior. It points out the importance of child engagement in the interaction process and provides evidence of a correlation between engagement and factors of valence and arousal in children with autism.

The study [53] aims to improve the strategies used in different social situations with the use of the robot-based intervention. The interventions aim to modify irrational beliefs (based on Albert Ellis’ therapy model) to teach adaptive behaviors in social situations associated with anger and sadness, and to reduce the intensity of negative emotions. The study showed that children with ASD from the robot-enhanced therapy group showed statistically significantly more rational beliefs, and that they had a lower level of emotional response intensity after treatment, compared with the usual group. The paper did not find any significant differences between the two groups regarding social knowledge and adaptive behaviors.

Another study proposed a framework to determine a child’s emotional state at any given point during an interaction to facilitate the delivery of robot-assisted tailored therapy interventions that can evoke a higher level of engagement from children with ASD [17].

The paper [20] presents a complex and comprehensive study of the human–robot interaction loop, especially focusing on child physiological response. This study pointed out interesting recommendations and challenges:

- Before automatic emotion recognition is performed, it is advisable to define a list of emotional states that would provide a value from the intervention perspective;
- Children with autism deficits in communication skills make it hard to apply classic methods of tagging emotional states;
- Physiological signals are continuously available and are not impacted by deficits;
- For the enhancement of the reliability of tagging, a clinical observer and a caregiver who knows the participant shall be included in the study;
- A preliminary phase might be added to train recognition models before the actual tasks to be monitored are performed.

The dissertation [31] presents a robotic platform that is used as a mediator in social interaction activities with children with special needs. The main purpose of this dissertation is to develop a system capable of automatic emotion detection through facial expressions and of interfacing with a robotic platform in order to allow social interaction with children with special needs. The system has two parts: (1) a Mirroring Emotion System synthesises human emotions through facial expressions online; the subsystem extracts the user facial action units and sends the data to the robot, allowing for online imitation, and (2) a Emotion Recognition System that is able to recognize human emotions through facial features in real-time.

Analyzing the variety of studies in the development of real-life applications of emotion recognition in autism therapy, some questions seem of the highest importance to be asked at the very beginning of each study:

- For what purpose are emotions recognized—is it to better understand the phenomena, or support intervention, or adjust technology (robot, app) behavior?
- In what way would emotion recognition help to develop skills in children with autism?
- The training of which skill would require automatic emotion recognition?
- Which emotions are of the primary interest? Perhaps the most frequently used set of six basic emotions is not the set that we shall be looking for, as practical studies point, rather, to engagement as a crucial affective state for the therapy effectiveness.

## 4. Discussion

Summing up the study, we were surprised by the fact that out of so many studies that dealt with emotion recognition in autism, we reduced it down to 50 papers (36 in the first search and 14 in the second one) included in the qualitative and 27 papers (17 in the first search and 10 in the second one) included in the quantitative analysis. There are several reasons for that. First of all, the domain is relatively young and, thus, the number of papers related to it is limited. This was partially proven by our second search, which revealed 10 relevant papers from only two years to be included in the quantitative analysis. Secondly, we excluded many interesting papers at first sight, but they described ideas or plans of studies that were not validated yet. Some papers turned out to be short communications only, and most of the papers were excluded due to no real participants’ involvement.

### 4.1. Answering Research Questions

The first research question regards the issues of distinct emotions that are automatically tracked in autism-related studies and the representation models of those emotions. The most common model of emotions used is Ekman’s six basic emotions set, but usually only a subset of them is actually recognized in a particular study. A valence–arousal model is also used in several studies. The observations are compliant with those by Rashidan et al. [9]—the study listed papers with emotions recognized (subsets of basic emotions as well as a single paper with a valence–arousal model), although no further analysis was performed. We additionally analyzed other emotional states addressed and the co-occurrence of states.

We noticed that some authors refer to emotional states from the basic set or other ones with their own labels, and those labels are not further defined from a psychological perspective. Some terms used by authors could be grouped. For example, happy, happiness, joy, and smile are hard to differentiate; therefore, we treated them as a group of joy-related emotions. Another example is fear-related emotions such as fear, anxiety, and trepidation. None of those terms is explicitly defined in the studies. Some states addressed in the studies are more attention-related than emotion-related (engagement, involvement), and that seems more of the real interest in studying children with autism (and not six basic emotions). An interesting concept of compound emotions, such as fearful surprise or happily disgusted, was also raised in the context of the meltdown crisis of a child; however, those were not automatically recognized and require further investigation. More detailed observations regarding emotional states addressed by the analyzed studies are provided in Section 3.2.1 and Section 3.2.2.

The second research question concerned observation channels that are used in emotion recognition in children with autism. We noticed that papers usually mix terms related to life activities observed, channels of observations, signals, and modalities analyzed. Authors frequently use those concepts interchangeably, for example, assuming that facial activity is connected with analyzing the RGB video channel only. We first defined the distinction between observation channel as a medium and modality as a type of information analyzed to obtain an emotional state estimate (see Section 2.1), and then analyzed the source as the triad of life activity–channel–modality. For example, we found out that facial expressions were tracked using facial features from RGB video (11 studies) or using spatial features from depth camera recording (2 studies), or using facial muscle tension from EMG sensors (1 study). The other review [9] lists channels analyzed for emotion recognition per paper, but using the triad is a novel approach introduced in our study.

The modalities that were analyzed by the papers in order to obtain emotional state included (with decreasing popularity, followed by the number of papers): facial expressions (14), skin conductance (10), heart rate (8) and heart rate variability (7), peripheral temperature (6), prosody of speech (6) and vocalisations (5), muscle tension (4), hand gestures (3), body motion (3) and body posture (3), head movements (2), respiration (2), eye gaze (1), and neural activity (1). The observation channels used included: video (RGB or depth), audio, EDA (electrodermal activity), BVP (blood-volume pulse) or ECG (electrocardiograhy), EMG (electromyography), and other physiological sensors as well as fMRI (functional magnetic resonance imaging). We also found an interesting paper that analyzed children’s drawings in order to recognize emotional states [47]. Section 3.2.3 reports more a detailed analysis, including the co-occurrence of modalities and a combination of modalities and emotional states recognized from them.

The third research question concerns techniques used in emotion recognition in children with autism, especially the classifiers or alternative approaches used. Diverse machine-learning methods are used (decreasing popularity, followed by the number of papers): SVM classifier (11), neural networks (7), k-nearest neighbors (3), linear discriminant analysis (2), random forest (2), fuzzy logic (1), and hidden Markov models (1). The dominance of SVM classifiers is compliant with the finding in [9]; however, the next methods differ, including euclidean distance clustering as the second one (3), Bayesian networks (2), and mean of correlation (2). There were also some studies that used off-the-shelf software for emotion recognition rather than classifiers trained for a specific study, e.g., a study on social robots used Noldus Face Reader software for the analysis of facial expressions [35]. We did not analyze how specific modalities were processed in order to obtain feature vectors, as each modality is processed with divergent transformations in a variety of combinations. We identified classifiers and their co-occurrence with specific emotions (see Section 3.2.4).

The fourth research question concerned techniques used for multimodal or multichannel emotion recognition (if more than one channel was used). The most popular was the unimodal approach (single channel and single modality). Fusion was used in 11 papers, with early fusion at the feature level being the most popular (10 papers), followed by late fusion (1 paper) and hybrid fusion (1 paper)—those do not sum up to 11, as one paper covered both early and hybrid fusion. We have also pointed out studies that used multiple channels and/or modalities, but separately analyzed and not integrated at any level (5 papers). Section 3.2.5 provides more details on fusion methods, including co-occurrence with emotions and machine-learning methods.

The study allowed us to answer the Research questions RQ1, RQ2, and RQ4 comprehensively. The third research question, RQ3, was partially addressed—we covered machine-learning techniques, but methods for signal processing would require a separate in-depth study.

### 4.2. Other Observations

Apart from quantitative analysis, some observations and lessons learned are also gathered. The most important observations could be summarized as follows.

- Participant groups used in the analyzed studies were relatively small and skewed in terms of representation of male/female and high-/low-functioning individuals. That might result in lower generalizability of the findings;
- Regarding the stimuli, various approaches were used, including emotions evoked via pictures, videos, and serious games, as well as natural interaction. The applied stimuli were frequently treated as labels, as the “ground truth” was hard to determine. Labels were also applied by self-assessment, assessment by therapist and/or caregiver, or as a combination of those. The problem of stimuli and labelling is known in affective computing; however, in autism-related and child-related studies, it is even more challenging, as children with autism might react to stimuli differently than typically developing children. Moreover, even qualified therapists might find it hard to assign a label to a child’s behavior;
- Physiological signals are most frequently used for the recognition of happiness versus a neutral state, which is surprising, as they reveal more information on arousal (intensity) of the emotional state rather than on valence (positive/negative dimension);
- All channels are prone to some disturbance, and information on a specific symptom might be temporarily or permanently unavailable. The disturbances might be divided into activity-based (depending on a task), child condition-based (depending on child deficits or current state), and setup-based ones (technical and contextual issues).

### 4.3. Validity Threats

Some papers could have been omitted due to the search of the keywords in the title only, and we are aware of that limitation of our study. We were considering alternative approache;, however, we wanted to search in multiple and diverse scientific databases/search engines to discover both technical (e.g., IEEE) and more medical/psychological (e.g., PubMed) studies. Consideration of searching in keywords and topic revealed that not all of the chosen set of search engines covers the two search fields (see Table 1 for further reference). Searching by all fields revealed much too many studies (over two hundred thousand) to handle. Search by titles allowed us to cover all of the search engines and to apply the search string to the same field in all of them. Some validity threats are also connected with the inclusion/exclusion procedure. There were three stages: tagging by title, tagging by abstract, and full-text reading. Four independent taggers performed tagging by title and by abstract. A single person read the paper in detail and qualified content for this study. The inter-rater consistency for tagging (Fleiss’ Kappa coefficient) was not very high (0.543 in the first search and 0.607 in the second one). The value indicates moderate consistency among raters and the reason for that could be that taggers had diverse perspectives—both technical and pedagogical. The consistency could also be lowered by the tagging scale (a 2–1–0 scale was used), and one might consider tagging with another scale more reliable. Moreover, a relatively high percentage of papers were excluded from the full-text reading stage and the main reason for exclusion on that stage was the lack of validation with real participants.

## 5. Conclusions

The paper provides a systematic literature review for the challenge of automatic emotion recognition applied in studying and training children with autism. Over 2000 papers were initially extracted from 7 search engines, finally including 50 papers in a qualitative and 27 in a quantitative analysis.

The study reveals some observations regarding observation channels, modalities, and methods used for emotion recognition in children with autism. Qualitative analysis revealed important clues on participant group construction and the most common combinations of modalities and methods. The study might be of interest for researchers who apply emotion recognition or enhance methods for affect classification in autism-related studies.

This systematic literature review revealed a number of challenges related to the application of emotion recognition to studies on children on the autism spectrum. Some good practices were also identified. There are several implications of the findings, both for science and practical applications. Further works might analyze diverse stimuli in autism and perhaps create a stimuli set dedicated for that special group, adjusted to its reactivity type. Moreover, multimodal approaches seem not to be explored enough, and perhaps more studies would reveal more practical results. Other issues of concern in further studies include: (1) the participants’ group construction taking into account sex, developmental age, and level of functioning; (2) the mixed (compound) labelling approaches; (3) creating datasets for further research—with measurement and label data related only to children with autism, or (4) which emotions are of real interest in the analyzed interaction rather than starting with a basic emotions model.

## Figures and Tables

**Table 1:** Number of results obtained for each database and search field.

		Search Field			
		All Fields	Keywords	Topic	Title
Database	ACM Digital Library	126	8	na	21
	Elsevier Science Direct	71,860	1278	-	106
					(96)
	IEEE Xplore	272	5	na	44
	Scopus	92,484	3960	-	561
	Springer Link	46,086	na	na	na
	Web of Science	10,615	na	8395	509
	PubMed	6999	na	3349	324
	Total	228,442	16,995		1565
					(1555)

**Table 2:** Papers describing recognized emotions groups.

Emotion Group	Number of Papers	Emotions—Notions Used
attention-related	6	engagement (3): [19,22,23]
		involvement (2): [21,24]
		attention (1): [25]
		bored (1): [25]
fear-related	16	fear (12): [17,26,27,28,29,30,31,32,33,34,35,36]
		anxiety (3): [20,22,25]
		trepidation (1): [37]
joy-related	21	joy (5): [21,26,27,28,29]
		liking (3): [19,20,22]
		happiness (12): [17,24,25,30,31,32,33,34,35,37,38,39]
		smile (1): [40]
		relaxation (1): [37]

**Table 3:** Number of papers describing studies recognizing one of the emotions in Ekman model or neutral emotion.

Emotion	Number of Papers	Papers
joy	21	[17,19,20,21,22,24,25,26,27,28,29,30,31,32,33,34,35,36,37,38,39]
anger	12	[17,27,28,29,30,31,32,33,35,36,38,39]
fear	12	[17,26,27,28,29,30,31,32,33,34,35,36]
disgust	7	[17,25,27,29,32,35,36]
sadness	16	[17,25,26,27,28,29,30,31,32,33,34,35,36,37,38,39]
surprise	7	[17,27,29,31,32,33,35]
neutral	15	[21,24,25,27,28,29,30,31,32,33,35,36,38,39,41]

**Table 4:** Ekman emotions enriched with neutral emotion recognized together.

Emotions Recognized Together	Number of Papers	Papers
joy	3	[19,20,22]
joy, neutral	2	[21,24]
joy, sadness	2	[21,37]
joy, fear, sadness	2	[26,34]
joy, disgust, sadness, neutral	1	[25]
joy, anger, sadness, neutral	2	[38,39]
joy, anger, fear, sadness, neutral	2	[28,30]
joy, anger, fear, disgust, sadness, surprise	1	[17]
joy, anger, fear, sadness, surprise, neutral	2	[31,33]
joy, anger, fear, disgust, sadness, neutral	1	[36]
joy, anger, fear, disgust, sadness, surprise, netural	4	[27,29,32,35]

**Table 5:** Studies corresponding to channels used in the process of emotion recognition.

Life Activity	Modality	Channel	Papers
movement	facial expressions	RGB video	[17,23,26,27,29,31,32,33,35,38,41]
		depth video	[29,31]
		images	[39]
		EMG	[40]
	body posture	RGB video	[23,42]
		depth video	[42]
	eye gaze	RGB video	[17]
	head movement	RGB video	[23,28]
	gestures	RGB video	[23,28,42]
		depth video	[42]
	motion	RGB video	[17,23,42]
		depth video	[17,42]
sound	prosody of speech	audio	[17,23,25,30,36,41]
	vocalization	audio	[17,23,25,36,41]
heart activity	heart rate	ECG	[19,20,21,22,43]
		BVP	[21,23,24]
variability	HRV	ECG	[19,20,22,43]
		BVP	[21,24]
muscle activities	muscle tension	EMG	[19,20,21,22]
perspiration	skin conductance	EDA	[19,20,21,22,23,24,25,37,43]
respiration	RESP intensity and period	chest size	[43]
thermal regulation	peripheral temperature	temperature	[20,21,23,34,43]
brain activity	neural activity	fMRI	[30]

**Table 6:** Emotions recognized in the Ekman and Russel models, enriched with neutral emotion corresponding to channels used in the process of emotion recognition.

Life Activity	Modality	Channel	Joy	Anger	Fear	Disgust	Sadness	Surprise	Neutral	Valence/Arousal
movement	facial expressions	RGB video	[17,26,27,29,31,32,33,35,38]	[17,27,29,31,32,33,35,38]	[17,26,27,29,31,32,33,35]	[17,27,29,32,35]	[17,26,27,29,31,32,33,35,38]	[17,27,29,31,32,33,35]	[27,29,31,32,33,35,38,41]	[17,23,35]
		depth video	[29,31]	[29,31]	[29,31]	[29]	[29,31]	[29,31]	[29,31]	
		images	[39]	[39]			[39]		[39]	
	eye gaze	RGB video	[17]	[17]	[17]	[17]	[17]	[17]		[17]
	head movement	RGB video	[28]	[28]	[28]	[28]		[28]		[23]
	gestures	RGB video	[28]	[28]	[28]		[28]		[28]	[23,42]
		depth video								[42]
	motion	RGB video	[17]	[17]	[17]	[17]	[17]	[17]		[17,23,42]
		depth video	[17]	[17]	[17]	[17]	[17]	[17]		[17,42]
	body posture	RGB video								[23,42]
sound	prosody of speech	audio	[17,25,36]	[17,36]	[17,36]	[17,25,36]	[17,25,36]	[17]	[25,36]	[17,23]
	vocalization	audio	[17,25,36]	[17,36]	[17,36]	[17,25,36]	[17,25,36]	[17]	[25,36]	[17,23]
heart activity	heart rate	ECG	[19,20,21,22]						[21]	[43]
		BVP	[21,24]						[21,24]	[23]
	HRV	ECG	[19,20,21,22]						[21]	[43]
		BVP	[24]						[24]	
muscle activity	muscle tension	EMG	[19,20,21,22]						[21]	
perspira-tion	skin conductance	EDA	[19,20,21,22,24,25,37]			[25]	[25,37]		[21,24,25]	[23,43]
thermal regulation	peripheral temperature	tempera-ture	[20,21,34]		[34]		[34]		[21]	[23,34,43]
brain activity	neural activity	fMRI	[30]	[30]	[30]		[30]		[30]	
respiration	RESP intensity and period	chest size								[43]

**Table 7:** Identified groups of emotions corresponding to channels used in the process of emotion recognition.

Life Activity	Modality	Channel	Attention-Related Emotions	Fear-Related Emotions	Joy-Related Emotions
movement	facial expressions	RGB video	[23]	[17,26,27,29,31,32,33,35]	[17,26,27,29,31,32,33,35,38]
		depth video		[29,31]	[29,31]
		images			[39]
		EMG			[40]
	body posture	RGB video	[23]		
	eye gaze	RGB video		[17]	[17]
	head movement	RGB video	[23]	[28]	[28]
	gestures	RGB video	[23]	[28]	[28]
	motion	RGB video	[23]	[17]	[17]
		depth video		[17]	[17]
sound	prosody of speech	audio	[23,25]	[17,25,36]	[17,25,36]
	vocalization	audio	[23,25]	[17,25,36]	[17,25,36]
heart activity	heart rate	ECG	[20,21,22]	[20,22]	[19,20,21,22]
		BVP	[21,23,24]		[21,24]
	HRV	ECG	[20,21,22]	[20,22]	[19,20,21,22]
		BVP	[24]		[24]
muscle activities	muscle tension	EMG	[20,21,22]	[20,22]	[19,20,21,22]
perspiration	skin conductance	EDA	[20,21,22,23,24,25]	[20,22,25,37]	[19,20,21,22,24,25,37]
thermal regulation	peripheral temperature	temperature	[20,21,23]	[20,34]	[20,21,34]
brain activity	neural activity	fMRI		[30]	[30]

**Table 8:** Recognized emotions and group of emotions corresponding to machine-learning techniques.

Emotions Recognized	NN	FC-M	SVM	RF	HMM	k-NN	LDA	FL
joy	[26,32,36,39]	[29]	[19,20,21,22,24,27,28,31,33,39]	[28,38]	[17]	[34]		[25]
anger	[32,36,39]	[29]	[28,31,33,39]	[38]	[17]			
fear	[26,32,36]	[29]	[27,28,31,33]	[28]	[17]	[34]		
disgust	[32,36]	[29]	[27]		[17]			[25]
sadness	[26,32,36,39]	[29]	[27,28,31,33,39]	[28,38]	[17]	[34]		[25]
surprise	[32]	[29]	[27,31,33]		[17]			
neutral	[32,36,39,41]	[29]	[21,24,27,28,31,33,39]	[28,38]				[25]
valence and arousal	[23,42]		[43]		[17]	[34,43]	[43]	
attention-related emotions	[23]		[20,21,22,24]					[25]
fear-related emotions	[26,32,36]	[29]	[20,22,27,28,31,33]	[28]	[17]	[34]		[25]
joy-related emotions	[26,32,36,39,40]	[29]	[19,20,21,22,24,27,28,31,33,39]	[28,38]	[17]	[34]		[25]

**Table 9:** Unimodal or multimodal approaches corresponding to machine-learning techniques.

Unimodal/ Multimodal	NN	FC-M	SVM	RF	HMM	k-NN	LDA	FL
single channel only	[26,32,36,39,40]	[29]	[27,31,33,39]	[38]		[34]		
multiple channels separately analyzed	[23,41,42]		[43]		[17]	[43]	[43]	
early fusion	[23]		[19,20,21,22,24,28]	[28]		[43]	[43]	[25]
late fusion			[43]			[43]	[43]	
hybrid fusion						[43]	[43]	

**Table 10:** Analysis of modalities versus recognized emotions in the Ekman and Russel models.

Unimodal/Multimodal	Joy	Anger	Fear	Disgust	Sadness	Surprise	Neutral	Valence and Arousal
single channel only	[26,27,29,30,31,32,33,34,35,36,37,38,39]	[27,29,30,31,32,33,35,36,38,39]	[26,27,29,30,31,32,33,34,35,36,35,36]	[26,27,29,31,32,35,36]	[27,29,30,31,32,33,34,35,36,37,38,39]	[27,29,31,32,33,35]	[30,32,33,35,36,38,39]	[34,35]
multiple channels separately analyzed	[17]	[17]	[17]	[17]	[17]	[17]	[41]	[17,23,42,43]
early fusion	[19,20,21,22,24,25,28]	[21,28]	[28]	[25]	[25,28]		[21,24,25,28]	[23,43]
late fusion								[43]
hybrid fusion								[43]

**Table 11:** Analysis of modalities versus identified groups of emotions.

Unimodal/Multimodal	Attention-Related Emotions	Fear-Related Emotions	Joy-Related Emotions
single channel only		[26,27,29,30,31,32,33,34,35,36,37]	[26,27,29,30,31,32,33,34,35,36,37,38,39,40]
multiple channels separately analyzed	[23]	[17]	[17]
early fusion	[20,21,22,23,24,25]	[20,22,25,28]	[19,20,21,22,24,25,28]

**Table 12:** Life activities, modalities, and channels analyzed versus unimodal or multimodal approaches.

Life Activity	Modality	Channel	Single Channel Only	Multiple Channels Separately Analyzed	Early Fusion	Late Fusion	Hybrid Fusion
movement	facial expressions	RGB video	[26,27,29], [31,32,38], [33,35]	[17,23,41]	[23]		
		depth video	[29,31]				
		images	[39]				
		EMG	[40]				
	body posture	RGB video		[23,42]	[23]		
		depth video		[42]			
	eye gaze	RGB video		[17]			
	head movement	RGB video		[23]	[23,28]		
	gestures	RGB video		[23,42]	[23,28]		
		depth video		[42]			
	motion	RGB video		[17,23,42]	[23]		
		depth video		[17,42]			
sound	prosody of speech	audio	[36]	[17,23,41]	[23,25]		
	vocalization	audio	[36]	[17,23,41]	[23,25]		
heart activity	heart rate	ECG		[22,43]	[19,20,21,43]	[43]	[43]
		BVP		[23]	[21,23,24]		
	HRV	ECG		[22,43]	[19,20,21,43]	[43]	[43]
		BVP			[24]		
muscle activity	muscle tension	EMG		[22]	[19,20,21]		
perspiration	skin conductance	EDA	[37]	[22,23,43]	[19,20,21,23,24,25,43]	[43]	[43]
respiration	RESP intensity and period	chest size		[43]	[43]	[43]	[43]
thermal regulation	peripheral temperature	temperature	[34]	[23,43]	[20,21,23,43]	[43]	[43]
brain activity	neural activity	fMRI	[30]				

**Table 13:** Modalities analyzed together.

Modalities	Number of Papers	Papers
HRV, skin conductance	1	[24]
head movement, gestures	1	[28]
prosody of speech, vocalization, skin conductance	1	[25]
facial expressions, prosody of speech, vocalization	1	[41]
body posture, gestures, motion	1	[42]
heart rate, HRV, muscle tension, skin conductance	4	[19,20,21,22]
heart rate, HRV, respiration, skin conductance, peripheral temperature	1	[43]
heart rate, HRV, skin conductance, RESP intensity and period	1	[43]
facial expressions, eye gaze, motion, prosody of speech, vocalization	1	[17]
facial expressions, body posture, head movement, gestures, motion, prosody of speech, vocalization, heart rate, skin conductance, temperature	1	[23]

**Table 14:** Times when the two modalities are analyzed together.

	bp	eg	hm	g	m	pos	v	hr	HRV	mt	sc	RESPiap	t
**fe**	2	1	1	1	2	3	3	1	0	0	1	0	1
**bp**		0	1	2	2	1	1	1	0	0	1	0	1
**eg**			0	0	1	1	1	0	0	0	0	0	0
**hm**				2	1	1	1	1	0	0	1	0	1
**g**					2	1	1	1	0	0	1	0	1
**m**						2	2	1	0	0	1	0	1
**pos**							3	1	0	0	2	0	1
**v**								1	0	0	2	0	1
**hr**									6	4	6	2	2
**HRV**										4	7	2	1
**mt**											4	0	0
**sc**												2	2
**RESPiap**													1

3. The following abbreviations are used: facial expressions—fe, body posture—bp, eye gaze—eg, head movement—hm, gestures—g, motion—m, the prosody of speech—pos, vocalization—v, heart rate—hr, muscle tension—mt, skin conductance—sc, RESP intensity and period—RESPiap, peripheral temperature—pt.

**Table 15:** Times when the two channels are analyzed together.

	Depth Video	EMG	Audio	ECG	BVP	EDA	Temp.	Chest Size
**RGB video**	1	0	3	0	1	1	1	0
**depth video**		0	0	0	0	0	0	0
**EMG**			0	4	1	4	2	0
**audio**				0	1	2	1	0
**ECG**					1	6	4	1
**BVP**						3	2	0
**EDA**							5	1
**temp.**								1

**Table 16:** Notions describing children used in papers.

Notions Used	Number of Papers	Number of Occurrences
autistic child\children	32	341
child\children with autism	49	474
child\children with ASD	35	642
child\children with ASC	2	29
child\children on autism spectrum child\children with autism spectrum	41	117
autism child\children	4	13
normal child\children	15	52
typically developing child\children	22	103
neurotypical child\children	2	5
